# Martian atmospheric hydrogen and deuterium: Seasonal changes and paradigm for escape to space

**DOI:** 10.1126/sciadv.adm7499

**Published:** 2024-07-26

**Authors:** John T. Clarke, Majd Mayyasi, Dolon Bhattacharyya, Jean-Yves Chaufray, Nicolas Schneider, Bruce Jakosky, Roger Yelle, Franck Montmessin, Michael Chaffin, Shannon Curry, Justin Deighan, Sonal Jain, Jean-Loup Bertaux, Erin Cangi, Matteo Crismani, Scott Evans, Sumedha Gupta, Franck Lefevre, Greg Holsclaw, Daniel Lo, William McClintock, Michael Stevens, Ian Stewart, Shane Stone, Paul Mahaffy, Mehdi Benna, Meredith Elrod

**Affiliations:** ^1^ Center for Space Physics, Boston University, Boston, MA, USA.; ^2^ LASP, University of Colorado, Boulder, CO, USA.; ^3^ LATMOS, CNRS UVS/UPS/SU, Guyancourt, France.; ^4^ Lunar and Planetary Laboratory, University of Arizona, Tucson, AZ, USA.; ^5^ Space Sciences Laboratory, University of California, Berkeley, CA, USA.; ^6^ California State University, San Bernadino, CA, USA.; ^7^ CPI Inc., Arlington, VA, USA.; ^8^ Space Science Division, Naval Research Laboratory, Washington, DC, USA.; ^9^ NASA Goddard Space Flight Center, Greenbelt, MD, USA.; ^10^ University of Maryland Baltimore County, Baltimore County, MD, USA.; ^11^ University of Maryland, College Park, MD, USA.

## Abstract

Mars’ water history is fundamental to understanding Earth-like planet evolution. Water escapes to space as atoms, and hydrogen atoms escape faster than deuterium giving an increase in the residual D/H ratio. The present ratio reflects the total water Mars has lost. Observations with the Mars Atmosphere and Volatile Evolution (MAVEN) and Hubble Space Telescope (HST) spacecraft provide atomic densities and escape rates for H and D. Large increases near perihelion observed each martian year are consistent with a strong upwelling of water vapor. Short-term changes require processes in addition to thermal escape, likely from atmospheric dynamics and superthermal atoms. Including escape from hot atoms, both H and D escape rapidly, and the escape fluxes are limited by resupply from the lower atmosphere. In this paradigm for the escape of water, the D/H ratio of the escaping atoms and the enhancement in water are determined by upwelling water vapor and atmospheric dynamics rather than by the specific details of atomic escape.

## Teaser

Observations of H and D in the upper atmosphere of Mars reveal their escape into space and a paradigm for the governing processes.

## INTRODUCTION

There is abundant evidence that Mars underwent an early wet period, with liquid water flowing across the surface leaving behind clear erosion patterns (*1*–*3*) and the presence of clay in the surface soil (*4*, *5*). This wet climatic period appears to have ended a little over 3 billion years ago, and the fate of that water has inspired great interest (*6*–*8*). To some extent, the water remained trapped in the crust as Mars cooled, and to some extent, it was dissociated into H and O atoms with many of the atoms escaping into space from the top of the atmosphere (*9*).

A key indicator of the total amount of water that has been lost into space is the present-day ratio of the isotopes deuterium and hydrogen (D/H) in the martian atmosphere (*6*, *10*). Assuming that Mars was formed from the same material as Earth, supported by the D/H ratio in ancient martian meteorites (*11*), the primordial water would have had a similar D/H ratio on the two planets. As hydrogen atoms escaped into space from the top of the atmosphere, the H atoms escaped faster than D atoms due to their lower mass. The remaining water would have gained an increased ratio of D/H, the magnitude of which indicates the total amount of water lost into space over geologic time (*8*, *10*). The D/H ratio in martian water today is roughly four to eight times larger (D/H ~ 0.001) than at Earth (*10*, *12*–*14*), consistent with the loss of a substantial amount of water from Mars early in its history based on models of the loss processes (*9*, *10*, *15*). With the present ambiguities about the physical processes that drive atmospheric escape, however, the depth of this primordial water is highly uncertain, with estimated values of a global equivalent layer (GEL) from tens to hundreds of meters. While martian water evolved over time, it is primarily atoms that escape into space; thus, an understanding of the physical processes controlling the atomic loss rates is key to extrapolating back over the lifetime of Mars.

Water in the martian atmosphere today originates at the surface, with the main reservoir of water frozen into the ground and the polar ice caps (*1*–*2*, *4*). A fraction of that water sublimates and moves through the atmosphere with the seasons (the exchangeable reservoir), generally circulating from pole to pole in gaseous form (*16*). Atmospheric water molecules are broken into atoms by ultraviolet (UV) sunlight and ionospheric processes, and atoms in the upper atmosphere with velocities above the escape speed may escape into space. This happens by both thermal (Jeans) escape and by nonthermal processes that provide excess energy to a fraction of the atoms (*17*, *18*). The total H loss corresponds to the amount of lost water because at Mars water is the dominant reservoir of hydrogen and deuterium (*6*, *19*). For oxygen, the case is more complicated because the great majority of the total atmospheric O is in CO_2_ and only ~0.1% is in H_2_O. This report concentrates on the study of hydrogen as a proxy for the escape of water.

The ratio of D/H in water in the lower atmosphere of Mars has been measured through remote sensing (ground-based, airborne, and space-based) infrared (IR) spectral observations (*20*–*22*) and via in situ mass spectrometer measurements (*12*, *23*). Values have shown evidence of seasonal and hemispheric changes by factors of 2 to 5 (*24*–*28*), and here, we assume a mean value of D/H ~ 0.001 in martian water in the lower atmosphere, which is roughly five to eight times greater than ocean water on Earth. Understanding the physical processes active today requires measuring the escaping H and D atoms in the upper atmosphere and comparing these numbers with the ratio in the water reservoir. The relationship between the observed enrichment in D/H in atmospheric water and the amount of water lost over time is governed by the fractionation factor *f*. Fractionation is the process by which the ratio of D/H in residual water increases with the loss of atoms to space, defined by a factor

$$f=\frac{\Phi_{D}/\Phi_{H}}{\left[\text{HDO}\right]/2\left[H_{2}O\right]}$$ (1)

where Φ is the global escape flux in atoms per second, and [HDO] and [H_2_O] are the number densities in molecules per cubic centimeter (*18*). This specifies the rates at which H and D atoms are escaping and relates them to the present elevated ratio of D/H in the water reservoir. This paper reports time series measurements of atomic D densities and escape rates in the upper atmosphere and thereby the fractionation factor measured over several martian years.

### Observations

H and D atoms in the martian upper atmosphere can be measured by resolving their UV Lyman-α emissions at high spectral resolution (<0.001 nm). Lyman-α emission is produced by resonant scattering of solar photons by H and D atoms. This light penetrates to a depth where it is absorbed by CO_2_, roughly 80 to 100 km altitude depending on the angle of incidence. The H and D Lyman-α emission lines have a wavelength shift (0.033 nm) corresponding to the small isotopic difference in energy levels between H and D atoms, and they both scatter photons in the spectrally broad solar Lyman-α emission line (*29*). Relating the emission brightnesses to densities requires the application of a radiative transfer (RT) model that accounts for multiple scattering of photons in the atmosphere. This leads to a degeneracy between derived values of the atomic density and temperature which has been carefully studied (*29*) and is further discussed in Materials and Methods. Another complication comes in relating local measurements to global values to derive the total loss rates. Measured values at each location on the planet have been converted to the values at the subsolar point and then integrated across the martian globe using assumed functional relations with solar zenith angle (SZA) (*30*).

**Fig. 1.**
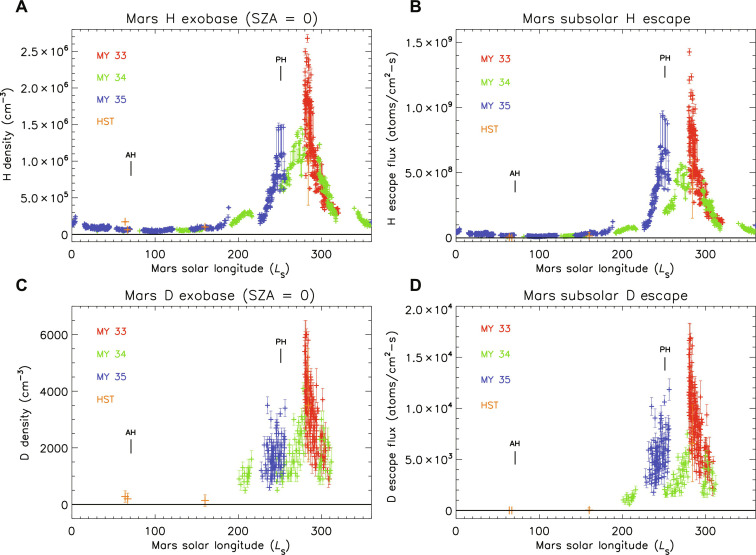
Martian upper atmospheric H and D densities and escape fluxes at the exobase and SZA = 0 with Mars solar longitude *L*_S_ measured over several MY. (**A** and **B**) Derived values for hydrogen; (**C** and **D**) derived values for deuterium. The MY 33 to 35 values are from MAVEN data, and for deuterium, they are upper limits except near perihelion (only detected values are plotted). These values are all for observations of the martian sunlit disc, and the periods of observation are listed in the Supplementary Materials. AH indicates the time of Mars aphelion, and PH indicates perihelion.

Observations of the H and D Lyman-α emissions from Mars have been performed with a dedicated echelle channel of the Mars Atmosphere and Volatile Evolution Imaging Ultraviolet Spectrograph (MAVEN IUVS) instrument (*31*–*33*) supplemented with a few observations using the Hubble Space Telescope (HST) Goddard High Resolution Spectrograph (GHRS) and Space Telescope Imaging Spectrograph (STIS) instruments (*34*–*36*). MAVEN IUVS in orbit about Mars has made observations with a high duty cycle since 2014, giving a complete picture of the seasonal changes but not detecting the D emission outside of martian perihelion periods when its density is high. Including a few higher sensitivity HST observations gives a good sampling of the variations over the full seasonal cycle on Mars, and one overlapping IUVS/HST observation from MY 34 shows good agreement in the results (Fig. 1). Both H and D emissions show large, repeatable seasonal changes in brightness and in atomic escape fluxes (*30*, *33*, *37*–*39*) as the Sun-Mars distance varies over the martian year.

Initial reports of the H and D emissions from the IUVS echelle data (*30*, *33*) were based on observations of their altitude profiles above the sunlit limb of Mars. This geometry permits modeling of the H and D densities with a high accuracy but requires long scans to build up the signal to noise. The limb geometry also introduces uncertainties due to the presence of interplanetary hydrogen (IPH) emission (*40*) above the limb and the presence of superthermal atoms above the exobase. This report presents observations looking down toward the sunlit disk, in which the signal is dominated by low-altitude emissions with the IPH blocked by the planet. This measures the full column of atoms down to ~80 km altitude that is most relevant to escape. The H emission is optically thick, while the D emission is optically thin, leading to different altitude profiles and contributions to the observed emissions, and the total columns derived from RT modeling are presented here. Density values are given for the exobase level from which escape occurs.

## RESULTS

The observed seasonal variations in H and D densities over Mars year (MY) 33 to 35 are plotted in Fig. 1. Mars’ seasons are dominated by the changing distance from the Sun and secondarily by the tilt of the planet on its axis, in contrast to Earth, so that the change in solar flux and temperatures are much larger. These observations covered a wide range of solar wind conditions and dust storm activity, yet the general trend of increasing densities around perihelion was observed each year. In addition to the seasonal changes, there are large and rapid changes in density around perihelion on slightly different dates in different martian years. These are most easily seen in the higher signal to noise H densities, and after careful testing, it has been determined that they are not due to changing observing geometry or assumed temperature. The increases on a timescale of a few weeks are faster than expected from estimates of the timescale for eddy and molecular diffusion of H_2_ to the upper atmosphere, while the decreases are consistent with estimates of the timescale for Jeans escape of thermal H into space (a few days near perihelion). Within the limits of the data, the temporal variations in D are similar to the observed trends in H. These large and rapid changes indicate that processes exist that can quickly supply atoms and quickly remove them from the upper atmosphere.

Martian H Lyman-α emissions exhibited large seasonal variations in brightness in prior observations with HST (*37*, *39*), Mars Express (MEX) (*38*), and MAVEN (*30*, *33*, *41*–*42*). Increases in water in the middle and upper atmosphere have been detected with similar seasonal variations with the MEX Spectroscopy for the Investigation of the Characteristics of the Atmosphere of Mars (SPICAM) (*43*–*44*) and the Trace Gas Orbiter atmospheric chemistry suite (ACS) and Nadir and Occultation for Mars Discovery instruments using IR solar occultations (*24*–*25*, *45*–*46*) and indirectly by the MAVEN Neutral Gas and Ion Mass Spectrometer (NGIMS) instrument (*47*). The seasonal increases in H and D reported here are consistent with the timescale for upwelling water (*46*, *48*–*52*) through large-scale motions of the atmosphere rather than a slow upward diffusion of molecular hydrogen. The observed decreases also require that both H and D be removed from the upper atmosphere on timescales of a few weeks or less. A strong global circulation pattern develops in the martian atmosphere in southern summer/perihelion (*16*), resulting in strong advection and transport in the middle and upper atmosphere. The observed changes support the importance of meridional dynamics in the upward flux of H_2_O and in the transport and escape of H and D, and this scenario is consistent with modeling (*51*–*53*).

To estimate the global escape fluxes of H and D, one can extrapolate the subsolar values across the planet by accounting for temperature and density variations with the SZA. The average global escape flux of thermal H near aphelion is 2 × 10^25^ atoms/s and near perihelion is 8 × 10^26^ atoms/s (column rates are given in Fig. 1). The Jeans escape flux is more sensitive to temperature than density. For example, during a space weather event, the H density decreased, while the temperature increased, leading to a net increase in the H escape flux (*54*). For the overall escape rates of H and D, it has been previously pointed out that H escape from Mars is source limited (*37*, *55*), that is, the time for atoms to escape from the top of the atmosphere is short compared with the rate of supply from lower altitudes. The rate of escape of thermal D is much slower, typically by a factor of 50 to 100, with a relatively small fraction of the atoms escaping throughout the martian year. Despite a considerable density of H_2_, because of its larger mass, the escape rate for H_2_ is much lower than for H atoms, and thermal escape of H_2_ does not contribute substantially to the total escape of hydrogen.

The upward mixing of water above the expected cold trap for condensation is due to strong global atmospheric circulation and turbulence occurring during southern summer, aided by increased heating from dust in the lower atmosphere and potentially including ice and water-coated dust particles (*49*, *56*–*57*). The repeated strong seasonal trend now observed every MY from MY 28 to MY 35 indicates that the process operates independently of the level of dust storm activity (*30*, *46*, *58*), while heating from dust storms also plays a role during some periods (*59*). A more detailed comparison of the H escape rates and individual dust storms has been presented (*42*). It is worth noting that no increase in H and D density was observed in the echelle data during the 2018 (MY 34) planet encircling dust event, although large increases in mesospheric water were observed. It appears that while the water was carried upward, this was not followed by a large injection of atoms into the thermosphere, and this will need to be explored in models of atmospheric dynamics.

**Fig. 2.**
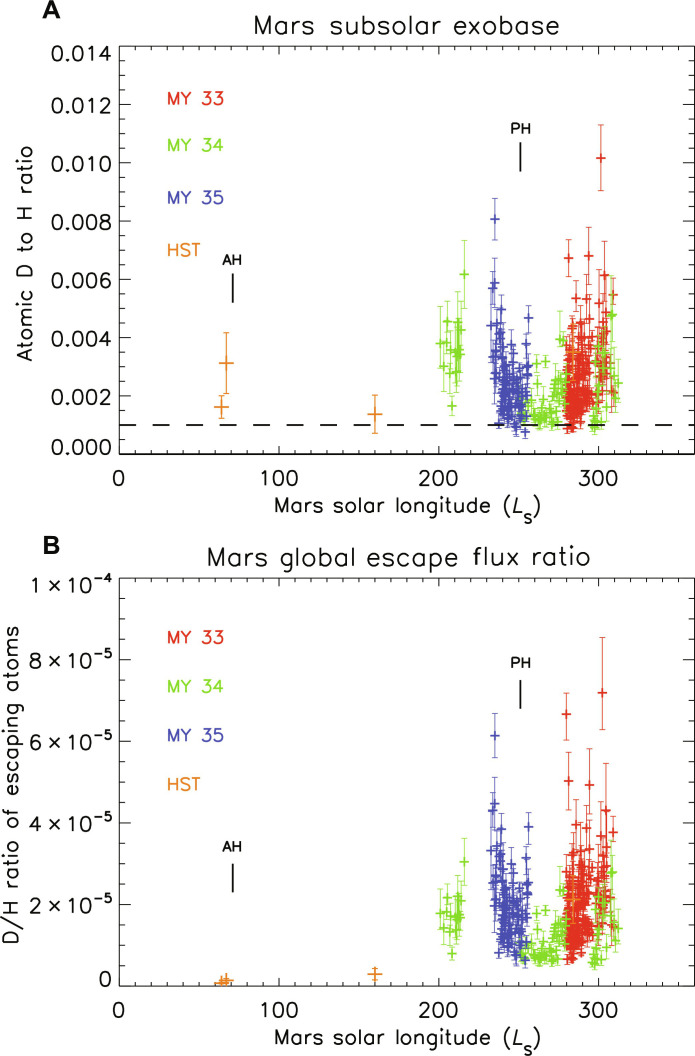
Density and escape ratios of deuterium to hydrogen in the upper Martian atmosphere. (**A**) D/H density ratio at 200 km at the subsolar point with solar longitude from the measured density values and a fit to temperature values measured with MAVEN NGIMS. The D/H value in martian water is ~0.001 for comparison (dashed line). (**B**) The global D/H ratio of the escaping atoms calculated for thermal (Jeans) escape versus solar longitude assuming the variations in density and temperature with SZA given in the text. The values are uniformly much lower than the ratio of D/H in martian water (~0.001) and the ratio of atoms in the upper atmosphere while also variable with seasonal changes. AH indicates the time of Mars aphelion, and PH indicates perihelion.

Combining the H and D densities gives the upper atmospheric D/H ratio with solar longitude (*L*_S_) over MY 33 to 35 (Fig. 2), and the values are consistently higher than the ratio in martian water. Near perihelion, the ratio of D/H atoms is generally a few times larger than the ratio in martian water, and there are indications of a seasonal change in the atomic D/H density ratio although this is at a lower statistical significance. A larger escape flux of the lighter H compared with D is expected to increase the D/H ratio in the thermosphere, and this difference is strongest when the densities are highest (near perihelion). At the measured temperatures of the thermosphere (roughly 200 to 280 K over MY 33 to 35), Jeans escape of H occurs on a timescale of days, but the rate for D is 50 to 100 times slower, not nearly fast enough to maintain the observed D/H ratio. The likely resolution of this difference comes from the added escape of superthermal atoms (both H and D), which modestly increases the H escape flux but markedly increases the D flux. As shown below, this process can maintain the observed conditions and may also explain the days to weeks changes in D density seen in the data.

The H and D atoms released from upwelling water can also have an enhanced D/H ratio due to the combined effects of condensation and photolysis, which have different rates for H and D. Estimates of this process suggest the D/H ratio is reduced by condensation and enhanced by photolysis (*27*, *60*–*61*). A higher rate of D escape compared with the thermal rate is still required to maintain the measured D/H ratio. A combination of these effects is likely to be responsible for the enhanced atomic D/H ratio in the upper atmosphere. Another line of reasoning is that because the escape of H is source limited, then the escape flux must be roughly equal to the upward flux of atoms from the lower atmosphere. On the assumption that the upward flux of atoms has a D/H ratio similar to that in water, we then also know the upward flux of D subject to alteration by different rates of condensation and photolysis (a factor of a few). Following this logic, the upward flux of D would exceed its escape rate by roughly two orders of magnitude in all seasons. The majority of D atoms must either return to the lower atmosphere or escape through other processes.

For the added escape process, there is strong evidence of superthermal H atoms in the upper atmosphere of Mars (*29*, *62*–*64*), and the same processes are expected to produce fast D atoms. Earlier work carried out before density changes and escape fluxes had been measured has noted that the primary process for loss of D could be fast atoms due to its low rate of Jeans escape (*6*, *35*, *65*). To estimate the loss of H and D from superthermal atoms, we can compare with an analysis of HST data performed with fits to the altitude profiles of the H emission. The fits were done first with a single elevated temperature and then with a measured temperature plus a superthermal component consistent with the known processes for the production of fast atoms (*66*). As an example, a population of D atoms with the same temperature (600 K) and fraction (2%) as superthermal H atoms (*29*) leads to a D escape flux 50 times higher than escape of the 280 K population alone. This can return the D/H ratio to the water value on a timescale of weeks in the absence of further upwelling of water, consistent with the measured trend between perihelion and aphelion. At aphelion, the measured D/H ratio is closer to the water value, implying that both D and H are lost rapidly as they diffuse upward, and in this season, the cold trap is expected to sequester water most rapidly. Once again, there is a surplus of D compared with the escape rate assuming an upward flux that is 0.001 times the measured upward flux of H. The expected reduced advection and transport at aphelion also support the superthermal atom scenario. Confirming this picture will require a better understanding of the production of superthermal atoms. While a small fraction of superthermal D atoms will be challenging to identify by measurement, identifying the processes responsible for superthermal H production will allow more accurate modeling of the number and velocity of fast D atoms because the two species are chemically similar.

Another line of inquiry is to compare the densities and escape fluxes of H and D atoms with the amount of upwelling water. The measured H column abundance above 80 km is ~3 × 10^13^ to 6 × 10^14^ atoms/cm^2^ varying over the year, with escape rates of 3 × 10^7^ to 10^9^ atoms/cm^2^-s. Water abundances of <1 (aphelion) to >50 (perihelion) parts per million by volume have been measured at 80 km altitude in the Trace Gas Orbiter ACS data (*45*). Assuming a 15-km atmospheric scale height, the corresponding column abundance of H atoms in water is <10^14^ to 10^16^ atoms/cm^2^. Around perihelion, the abundance of H in water is nearly 100 times greater than the measured column of H atoms. Near aphelion, the upper limit to water is comparable to the H column, but it is likely that the actual amount of water is below the upper limit set from H_2_O observations. Overall, this implies that most of the water that rises to 80 km is recycled into the lower atmosphere, consistent with photochemical models (*67*–*69*), and that only a small fraction of the H and D atoms from the water diffuse upward and escape. This supports the importance of lower atmospheric dynamics in the determination of escape fluxes (*53*).

Returning to the fractionation factor, historically, there have been large changes in solar insolation and the global climate of Mars due to obliquity and precession changes, the equivalent of Milankovitch cycles (*70*–*71*). Thus, conditions in the past have differed greatly from those at present. An advantage of applying the fractionation factor to these changes in the history of water loss is that the denominator represents an integrated value of exchangeable water over the history of Mars. For the numerator, how do the measured values relate to the global escape of water today? Near aphelion, the upper atmosphere is dry with low escape rates for both H and D, and the D/H ratio implies that the escape of both species is source limited by the slower upward diffusion of H- and D-bearing species. By far, the greatest loss to space and potentially the higher fractionation occur near perihelion. Another point is that, in this scenario, where superthermal processes dominate the escape of D atoms, the fractionation factor can vary considerably depending on the fraction of D atoms that escape at different times of year.

**Fig. 3.**
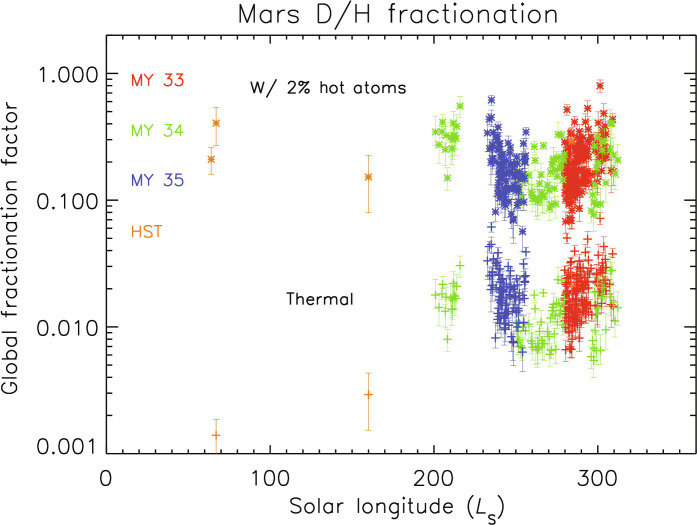
D/H fractionation factor calculated for two cases. Bottom dataset is for thermal (Jeans) escape alone, and top dataset includes 2% hot atoms at a temperature of 600 K. The need for superthermal atoms is discussed in the text, and this plot shows the large changes in fractionation factor when hot atoms are included.

In Fig. 3, the values of *f* derived from the data are plotted for two cases: one assuming thermal escape with the temperature of the CO_2_ atmosphere and the other including 2% hot atoms consistent with prior modeling of H altitude profiles. These two cases are intended to show the feasible range of values of *f* since the escape rates of hot atoms are not yet well determined. Note that, in both cases, *f* is not a constant with time and may vary on short timescales. In each case, the point-to-point variability exceeds the error bars; this is consistent with prior observed H and D profiles differing from the long-term average profile assumed in the derivations. The variability seen in the figure ultimately could be driven by spatial and/or temporal variations in atmospheric dust content, water vapor content, coupling in circulation between the lower and upper atmosphere, external forcing from solar particles, or other processes. In the case of source-limited escape of both H and D (upper values), the fractionation factor approaches large values in the range 0.1 to 0.6 depending on the other fractionation processes in the middle atmosphere. Earlier work using a one-dimensional photochemical model of D and H loss gave a value for *f* of 0.32 (*6*). With an updated analysis including additional chemical and dynamical processes, a value of less than 0.01 was calculated for thermal escape alone (*69*), similar to other estimated values (*35*). The need for superthermal atoms to explain the short timescale changes in atomic density supports the higher values of *f* corresponding to the loss of large volumes of water to space. Further work will be needed to pin down the fractionation factor and, in particular, the contribution of superthermal D atoms for an accurate extrapolation of the past water history of Mars.

Relating the fractionation factor to the original GEL of water on Mars requires added assumptions about the depth of the water reservoir that is exchanging vapor with the atmosphere and how much water is frozen into the crust without exchanging, since fractionation applies only to the water that is exchanging with the atmosphere (*9*, *72*–*73*). Overall, to extrapolate back in time, one must identify the dominant processes for atomic escape. The scenario presented above indicates that thermal Jeans escape of H and nonthermal escape of D are dominant throughout the year, even with large differences in densities and sources. An updated paradigm for escape of H and D is that both species escape from Mars rapidly throughout the year, and the differentiation in D/H atoms in the upper atmosphere is determined mainly by large-scale processes in the lower atmosphere. In this paradigm, the same processes are likely to have dominated the escape of D and H over a wide range of climatic conditions, lending support to a long-term high value for the fractionation factor. Values of *f* larger than 0.32, which has previously been used (*6*, *35*), appear at times in Fig. 3 and within the uncertainties *f* is not constant. Higher values of the fractionation factor implied by a high escape rate of D correspond to a primordial H_2_O GEL hundreds of meters thick on Mars (*74*), but this depth is sensitive to the value of *f*.

We can estimate the amount of water lost through time assuming thorough mixing between the atmosphere and the non-atmospheric exchangeable reservoir of water (mainly the polar caps) and assuming a value of *f* that was constant through time. For low *f* (e.g., less than 0.01, in which case little or no D escapes), the limiting value of the amount of water lost is about six times the current exchangeable reservoir. For a 20-m GEL of water ice in the polar caps today (*7*), this translates to ~120 m lost through time. For a value of *f* = 0.3, the amount lost is ~300 m GEL. More complex scenarios of exchange involving other sinks for H and D from water (*8*, *9*, *75*) would increase this value. Overall, the results presented here offer strong supporting evidence for a warm and wet period with an abundance of water on early Mars and a large amount of water loss into space over the lifetime of the planet.

## DISCUSSION

The densities of H and D atoms in the upper atmosphere of Mars and their rates of escape into space have large seasonal variations. The densities near perihelion (southern summer) are 5 to 20 times higher than near aphelion, and the escape rates are 10 to 100 times higher. The high H and D densities near perihelion are consistent with the trend in measured water increases in the middle atmosphere, with an annual cycle persistently driven by seasonal atmospheric dynamics and a variable contribution from heating by atmospheric dust. By the numbers, it appears that most of this water remains in the atmosphere, possibly mixing back down into the lower atmosphere by the same dynamical processes that raise water into the middle atmosphere. The D/H ratio of the escaping atoms is a few times higher than the ratio in water with a possible increase near perihelion.

H atoms in the upper atmosphere are lost rapidly by thermal escape in all seasons, and the escape flux is limited by the amount diffusing upward from the lower atmosphere so that the escape flux effectively equals the upward flux. The D escape flux from thermal escape is negligible, in which case an upward flux with the water-based D/H ratio would result in a large surplus of D in the upper atmosphere. Enhanced escape of superthermal D atoms appears to be required to restore the D/H ratio to the measured equilibrium with H near aphelion and to be consistent with observed faster changes in D density near perihelion. In this scenario, the fractionation factor becomes much larger, consistent with a large primordial reservoir of water on Mars. We consider this to be the likely scenario, while more work is needed to understand the physical processes responsible for superthermal atoms and their escape.

When H and D atoms escape to space on timescales of days to weeks, the escape fluxes are limited by the source rate of new atoms from upwelling water. With source-limited escape of both species, the details of the escape processes are less important than the source rate, and the differentiation of D and H atoms moving upward may be dominated by large-scale processes in the lower and middle atmosphere. This is an updated paradigm for understanding the history of water escape from Mars, in the sense that the escape fluxes of both H and D atoms and their fractionation factor are controlled not by the details of the escape processes but by the source rates from the lower atmosphere. The trends reported here represent substantial progress toward understanding the physical processes that govern the escape of hydrogen into space at Mars and our ability to relate these to the isotopic fractionation of D/H and the depth of primordial water on Mars.

## MATERIALS AND METHODS

### Observational data

The IUVS instrument on the MAVEN spacecraft includes a dedicated echelle spectral channel that separately measures H and D Lyman-α emissions. The minimum detectable line emission is ~100 Rayleigh brightness, which limits the detection of D emission to periods around perihelion. Observations have been made on a regular basis since MAVEN arrived at Mars in September 2014, and the data here cover parts of MY 33 to 35 (in MY 35, the MAVEN orbit was altered to use the spacecraft as a telecom relay for surface probes, and later data are not included). The few HST observations are from MY 20, 23, 25, and 34. The overlap of the MY 34 IUVS and HST measurements demonstrate the consistency of the measurements between the two missions. The combined coverage provides a good measure of long-term changes and repeatability from year to year, compared with a small number of HST observations from different years. The MAVEN data from MY 32 are excluded since the observing sequences early in the mission used short exposures and are challenging to compare with later data.

The times and solar longitudes of the IUVS echelle observations covered a range of SZA, and the data in this paper are limited to SZA < 75°. The observations in this paper are limited to the data taken before the MAVEN orbit was altered for operation as a telecom relay for consistency. The orbit of MAVEN about Mars slowly precesses, yielding observations whose geometry is continuously evolving, and observations of the dayside atmosphere with direct solar illumination within 75° of the subsolar point are included in this paper. Before the data were plotted, each measurement at a given SZA was related to the corresponding value at the subsolar point with assumed variations of temperature and density with SZA (*30*, *42*).

Three HST observations of Mars around aphelion and one close to perihelion were obtained with two high spectral resolution UV instruments, the GHRS and the STIS. The data can be accessed from the Space Telescope Science Institute Mikulski Archive for Space Telescopes archive, and the aphelion observations have been reported previously (*34*–*36*). To be consistent with the modeling presented in this paper, the published brightness values have been reanalyzed with the same RT code applied to the IUVS data (*58*). The two GHRS observations were with a single aperture roughly 2 arc sec square, with the first observation (9 to 25 May 1991) nadir-pointing and second (20 to 21 January 1997) pointed at the sunlit limb. Both were near Mars aphelion with *L*_S_ values of 64 and 67. The signal-to-noise ratio and significance of detection of the D emission in the two datasets are comparable, and the updated density values are different from what was originally reported. In particular, the second observation observed the sunlit limb of the planet, where the observed path through the atmosphere changes rapidly with location, and the original authors assumed that there would be a strong limb brightening in the D emission which resulted in a very low derived HD density (*35*). A large number of limb scans with the MAVEN IUVS echelle have shown that such a large limb brightening does not exist (*30*, *33*), presumably due to variations in the atmosphere along the line of sight; thus, the new analysis of those data derives a much higher density of D that is comparable to values from the other HST observations.

The STIS observations were with a long aperture that covered the disk of Mars from side to side, adding the spatial distribution of the emission across the sunlit hemisphere to help constrain the modeling. With the benefit of the updated RT model and observations of the center to limb profiles of both the H and D emissions with the MAVEN IUVS echelle data, we obtain more accurate values for the densities. The first STIS observation (9 to 14 May 2001, *L*_S_ of 160) was intermediate in season and density values between the GHRS and IUVS data. The final STIS observation was at *L*_S_ of 284 during MY 34 and close in time to IUVS observations. The measured brightnesses for H and D emissions with STIS were consistent with the IUVS data within 20%, and the absolute sensitivity calibration of the IUVS echelle data was derived in part from comparison with HST measurements (*40*). Temperatures for the times of the HST observations were estimated using the Laboratoire Meteorologie Dynamique global circulation model (*76*). By coincidence, the two GHRS observations were performed at similar *L*_S_ values but years apart at different levels of solar activity. Temperatures and atomic densities were higher in the period of higher solar activity during the 1991 observations compared with 1997. The MY 34 STIS observation near perihelion shows the consistency of measurements and derived densities between HST and IUVS.

### Deriving H and D densities from Lyman-α emissions

Multiple scattering of photons in planetary atmospheres is modeled using an RT code to derive density from measured emission brightness. Prior flyby observations of Mars by the Mariner (*55*) and Rosetta (*77*) missions are examples of this method. Incident solar Lyman-α intensities are taken from measurements by the MAVEN Extreme Ultraviolet Monitor instrument (*78*). The RT codes used in this work are described in (*62*, *66*), and the two models give consistent results.

In the case of the Martian upper atmosphere, the D Lyman-α emission is optically thin, while the H emission is optically thick, so that the D densities are nearly linearly proportional to the emission brightness while H densities are not. In the model, the brightness will increase with both increasing density (more atoms scattering the incident photons) and with increasing temperature (large Doppler spread of atom velocities scatters more of the broad solar emission line). This introduces a degeneracy of solutions for a given brightness. As in past work, we have used independent values of the neutral temperature (measured for IUVS and modeled for HST) and then solved for the density.

Another complication lies in the known variations in upper atmospheric density and temperature with SZA and local time. The main factors that determine the temperature are the input solar extreme UV (EUV) flux and radiative cooling by CO_2_, both of which vary across the planet by large factors. It is expected that there may also be large variations in atomic density from varying production rates, loss rates, and dynamics. Here, we have used the model of (*58*) to derive the density from the measured brightness at the point of observation and then converted to the subsolar value based on the SZA. The variation of temperature with SZA is provided by a fit to a large sample of data from the MAVEN NGIMS instrument (see below), and the variation of density with SZA is taken to be the function in (*79*) based on calculated dynamical motions of H atoms in the exosphere. The global values for escape rates depend on the nature of these assumed SZA dependencies, and as more data come down from MAVEN, we hope to be able to test and potentially improve this approach.

### Determination of upper atmospheric temperature

Knowing that the escape fluxes are highly sensitive to the assumed temperature, the thermospheric temperatures measured by MAVEN have been examined. Local temperatures have been measured by the MAVEN NGIMS instrument (*80*), and mean values along a line of sight column have been measured by the MAVEN IUVS instrument (*81*). These data are both based on measurements of the density scale height, from which a temperature value is derived, and both show large and rapid temperature changes with location and time. The local density measurements by NGIMS include large amplitude gravity waves that do not represent the molecular temperature. For the IUVS data along a long column of atmosphere, these waves are somewhat averaged out but still include uncertainties from atmospheric turbulence. For the purposes of computing escape fluxes, one can average over longer times to obtain representative thermal values and then calculate the changes over a Martian year. The smoothed curve through the NGIMS temperature measurements that has been used in this paper is plotted in figure 3 of the referenced paper (*42*). These values are appropriate to long-term density and escape flux estimates while averaging over short-term variations. The sensitivity of escape flux to temperature for H and D is discussed below under the “Sensitivity of escape fluxes to temperature and density” section. The primary energy input to the upper atmosphere is the solar EUV flux, which varies by 1/*R*^2^ (*R* = distance to the Sun) between aphelion and perihelion in addition to intrinsic changes. The curve that has been fitted to the NGIMS data has a lower amplitude than 1/*R*^2^ as expected from the energy balance by IR radiative cooling by CO_2_ (*80*) and is consistent with model fits to the data in (*82*).

### Assumptions on altitude profiles of density

**Fig. 4.**
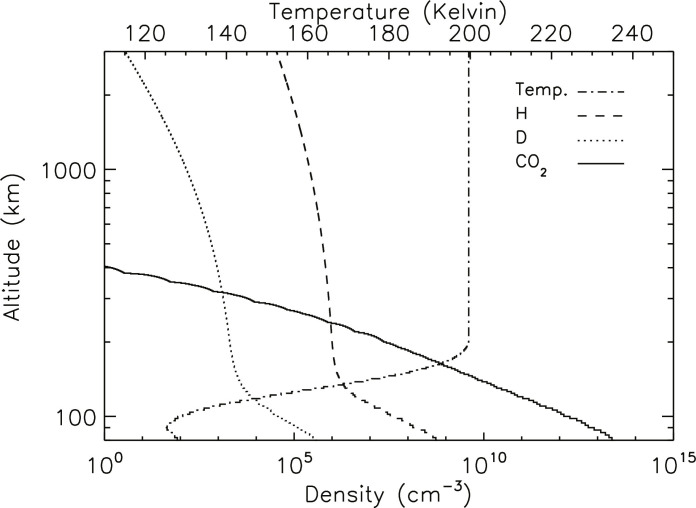
Altitude profiles of temperature and CO_2_, H, and D densities. Densities are in number per cubic centimeter, and temperatures are in Kelvin for one MAVEN orbit in MY 34 near *L*_S_ of 270 under the assumption of eddy and molecular diffusion at all altitudes.

In all the reported observations, the brightness of the H and D Lyman-α emissions depends on resonant scattering down to an altitude of ~80 to 100 km, where photoabsorption by CO_2_ dominates over scattering. In reality, the level where CO_2_ becomes optically thick to Lyman-α emissions varies in altitude as the temperature changes, while in the RT model, it is parameterized as an altitude. Setting the base at 80 km includes the dominant fraction of Lyman-α photons that can scatter solar emission across a range of temperature values. Relating measured brightness to density depends on the assumed altitude profiles of density for both H and D, which are not directly measured. Most of the scattering takes place at low altitudes in this column due to the higher densities. At altitudes above ~120 km, the density profile is determined by a combination of eddy and molecular diffusion, with the latter dominating at higher altitudes. Between 80 and 120 km, there is more uncertainty in the density profile of atomic species; therefore, we have tried a range of assumptions to learn the effect this would have on the derived density at the exobase. Three cases have been run through the RT model: (i) constant mixing ratio of H and D with altitude (an effective lower limit to the densities), (ii) a model profile based on atmospheric chemistry, and (iii) pure eddy and molecular diffusion. Comparing the three cases, the density numbers change, but the general trends in temporal variations and escape rates are retained. We report here values for case (*3*) containing diffusion alone (*83*). Examples of the altitude profiles of density for H and D are plotted in Fig. 4.

It is not possible with the available data to determine how the actual D and H profiles deviate point by point from the assumed average profile or to separate out short-term (subseasonal), seasonal, or year-to-year variability. The average values of *f* will be accurate to the extent that the profiles of D and H assumed in the analysis represent a long-term average. As examples, one can compare the average *f* values for the perihelion season and for the aphelion season, given the large variation in both D and H abundances between the two. The average value for thermal escape is about 0.005 during aphelion and about 0.02 during perihelion. Including nonthermal escape driven by the 2% hot atoms, both the aphelion and perihelion averages are about 0.2. In addition to these average values, there are substantial short-term changes in *f* resulting from measured changes in the D and H emission brightnesses. The similarity of the numbers that include hot atoms to the value calculated in (*6*) is probably coincidence, given that our current understanding of the processes and properties involved differs from the assumptions in that work. With our limited understanding of the role of nonthermal escape processes, it is not possible at this time to determine empirically a unique average value for “*f*” or of the processes controlling it.

**Fig. 5.**
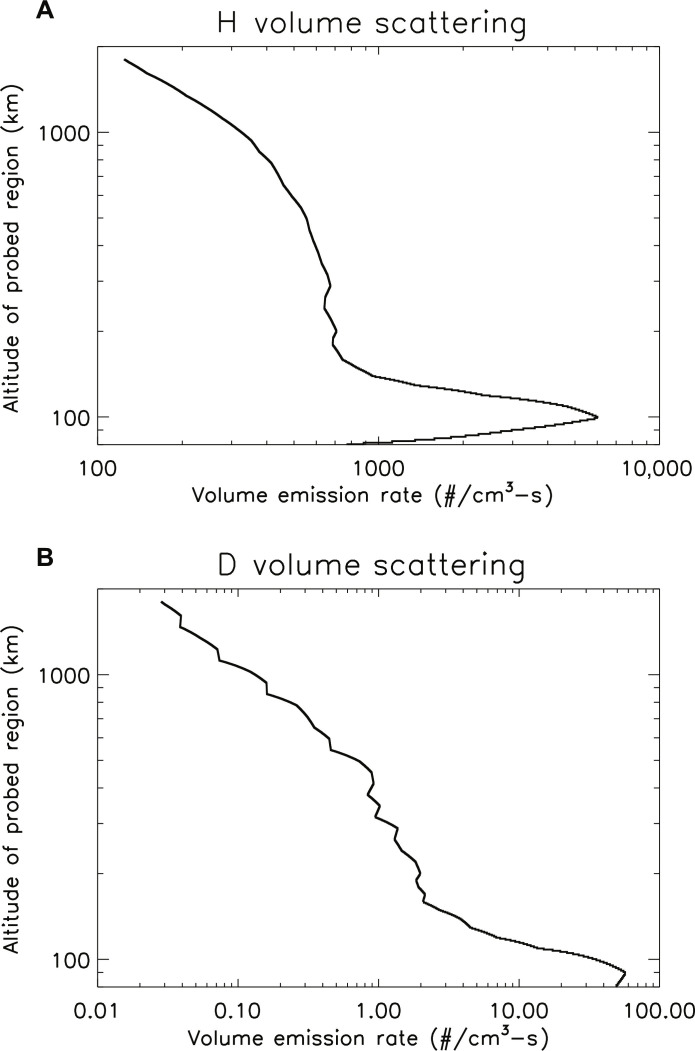
Number of scatterings with altitude for the H and D Lyman-α emissions. The values for (**A**) hydrogen and (**B**) deuterium are from the RT models assuming the model atmospheric profile in Fig. 4 and the observed brightnesses from the corresponding observation. The emissions that are observed are the integrated columns of photons down to ~80 km altitude.

Another important factor in interpreting the data is the altitude range that is sampled in the derivation of density from brightness in the RT modeling. This paper reports total columns of H and D above 80 km altitude, but the different optical depths of the H and D Lyman-α emissions mean that the derived densities represent different sampling in altitude due to the much larger number of scatterings of the optically thick H emission. As an example, Fig. 5 shows the number of scatterings by H and D atoms with altitude for the model atmosphere presented in Fig. 4. Between 80 and 100 km, the actual profiles may differ from the ones used in the model due to chemistry, and near 120 km, the density profiles depend on the chosen eddy diffusion profile and also the nonthermal D escape not included in the model. Above ~150 km, we do not expect substantial differences.

We have run another test case to determine the sensitivity of the derived column densities to variations in the altitude of the base of the column. Since CO_2_ is the dominant constituent of the atmosphere, it is tightly constrained by hydrostatic equilibrium, and its vertical extent depends on the local temperature in a well-understood manner. The altitude distribution of CO_2_ density and its changes have also been well studied by the MAVEN NGIMS and IUVS measurements (*80*, *81*). To learn more about the uncertainty in the atomic columns, we have run test cases with different values of *T* to determine the sensitivity of the total D column density and where the effective bases of those columns appear in altitude (tau CO_2_ = 1 refers to CO_2_ photoabsorption of incident solar Lyman-α)

$$\begin{array}{c} {\text{CO}}_{2}\;\text{nominal}\;\left(\text{tau}\;{\text{CO}}_{2}=1\;\text{at}\;80.7\;\text{km}\right)==>D\\ \;\text{column to}\;80\;\text{km}=2.89\times 10^{11}\;{\text{cm}}^{-2} \end{array}$$

$$\begin{array}{c} {{\text{CO}}_{2}}^{*}e^{+1}\;\left(\text{tau}\;{\text{CO}}_{2}=1\;\text{near}\;87.5\;\text{km}\right)==>D\\ \text{column to}\;80\;\text{km}=2.05\times 10^{11}{\text{cm}}^{-2} \end{array}$$

$$\begin{array}{c} {{\text{CO}}_{2}}^{*}e^{-1}\;\left(\text{tau}\;{\text{CO}}_{2}=1\;\text{near}\;73.8\;\text{km}\right)==>D\\ \text{column to}\;80\;\text{km}=6.56\times 10^{11}{\text{cm}}^{-2} \end{array}$$

Similar changes are seen in the H density values. These numbers show a variation in column density of a factor of 2 to 3 for this range in temperature, under the assumption that the H and D columns remain fixed while the CO_2_ density varies. This is unrealistic, since if the temperature varies, then H and D move up or down along with the CO_2_ and the column densities would change by smaller amounts; therefore, these values represent upper limits to the changes in the D column. It is important to keep in mind that the RT modeling integrates the vertical columns in density for both emissions, and for both species, the total columns of atoms contribute to the observed emissions.

### Excess D in the upper atmosphere resulting from thermal escape

For thermal Jeans’ escape, it has been found that H escapes much faster than D, which would lead to an increase in D in the upper atmosphere. The magnitude of this increase can be calculated by assuming that the source of H and D atoms is H_2_O molecules in the ratio 0.001 measured in lower atmospheric water. If the H atoms escape to space in a week or two, then the upward flux of H is effectively equal to the escape flux. From the assumed water source ratio D/H = 0.001, we then also know the upward flux of D and can calculate the thermal Jeans’ escape flux from the measured density and temperature. The resulting ratio of upward D flux to escaping D flux ranges from factors of tens to hundreds.

From these numbers, the upward flux of D greatly exceeds the escape flux throughout the year at Mars, raising the question of the fate of these atoms. There are two possible solutions, the excess D atoms can go up (escape to space by a nonthermal process) or go back down (diffuse into the lower atmosphere). For the latter case, the escape of H and downward flux of D would increase the ratio of D/H in the lower atmosphere, and one can estimate the timescale on which the overall D/H ratio in water would become enhanced by the factor of ~5 measured today. Note that both H and D should be subjected to any downward mixing, but as long as the timescale for H escape is a few days, this will dominate over a downward flux. We take the exchangeable column of water (the amount that exchanges between the surface and atmosphere over a Martian year) to be 10 precipitable microns (*8*) or a vertical column of 3.3 × 10^19^ molecules/cm^2^. For a long-term average H escape flux of 10^8^ atoms/cm^2^-s, the time to lose 80% of the H atoms (corresponding to an increase in D/H by X5) is ~20,000 years. Fresh H_2_O from a surface reservoir could replenish the exchangeable volume with orbital and precession changes, but the Martian Milankovitch cycles are thought to operate on timescales of 10^5^ to 10^6^ years. The other caveat to this rough estimate is that in addition to the seasonal changes, additional water from the polar caps could be exchanging with the atmosphere on a timescale shorter than 20,000 years. From this estimate, it appears unlikely but not impossible that the excess D could be recycled into the lower atmosphere.

Note that these numbers refer to a simplified case in which it is assumed that the numbers of H and D atoms that move up through 80 km altitude equally escape and are equally affected by atmospheric mixing. The initial case assumes that all upward moving atoms escape to space, whereas one expects a substantial number to be remixed below 80 km. The final result, however, shows such a large ratio of upward flux to escaping atoms for D that the main conclusion appears to be relatively insensitive to these assumptions. A more detailed calculation including atmospheric mixing and vertical motions would shed light on this.

### Sensitivity of escape fluxes to temperature and density

The Jeans’ process refers to the escape of atoms in the atmosphere at thermal equilibrium (a Maxwell-Boltzmann velocity distribution) that exceed the escape speed from a planet’s gravity. The expressions for Jeans’ escape flux and other key parameters are

$$\text{Escape flux}=\frac{\mathrm{nu}}{2\sqrt{\pi }}\;e^{-\lambda }\left(\lambda +1\right)$$ (2)

$$u=\sqrt{\frac{2\mathrm{kT}}{m}}\;\lambda =\frac{\mathrm{mgr}}{\mathrm{kT}}\;v_{\text{esc}}=\sqrt{2\mathrm{gr}}$$ (3)

where *n* is the number density (atoms per cubic meter) at the exobase (~200 km), *u* is the most probable speed, *k* is the Boltzmann constant, *m* is the atomic mass, *g* is the acceleration due to gravity, *v*_esc_ is the escape velocity, and *r* is the distance to the planet center. Values for H escape are divided by two to take into account the depletion of the high velocity tail of the distribution by escaping atoms due to H diffusion in a CO_2_ atmosphere (*18*). The escape rate of thermal D is sufficiently slow to not require this correction, but it is applicable to superthermal D. Note that in calculating the fractionation factor, it is important to add up all the escaping atoms over the globe and take their ratio, leading to the requirement for an assumed variation of density and temperature with SZA.

**Fig. 6.**
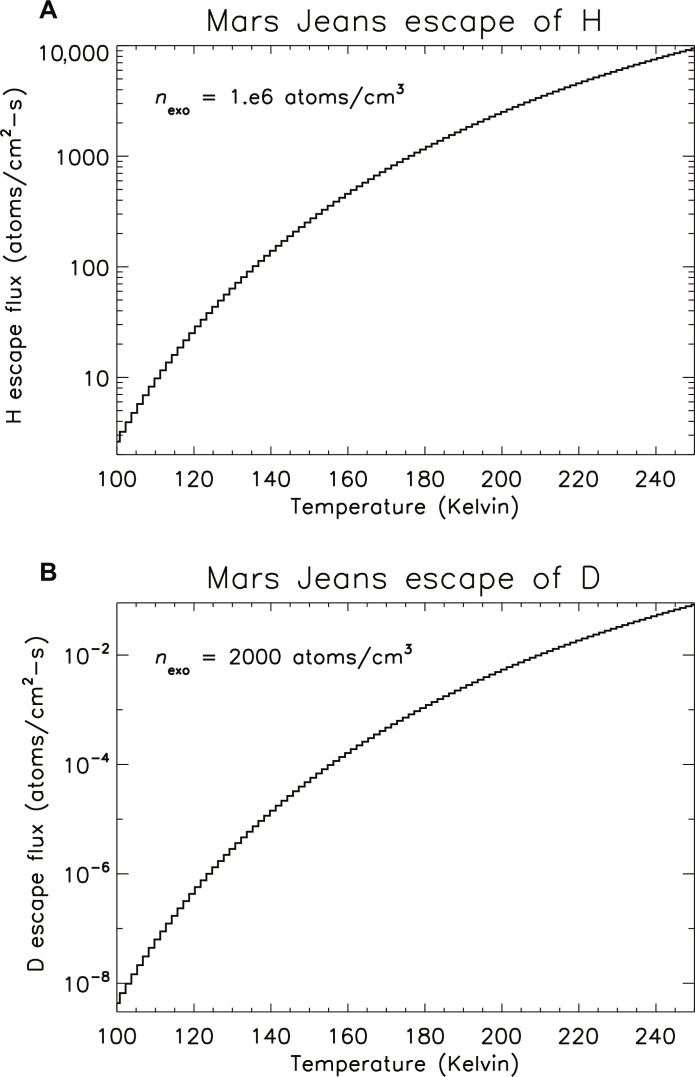
Escape fluxes of H and D atoms by the Jeans process at Mars versus temperature. The values for (**A**) hydrogen and (**B**) deuterium assume a Maxwell-Boltzmann velocity distribution. At the Martian exobase, atoms that exceed the escape speed of 4.87 km/s may be lost into space depending on their trajectory. The D escape flux is much smaller than for H in part due to lower density but mainly due to the heavier mass.

The escape flux is proportional to the atomic number density, while the dependence on temperature is nonlinear. To illustrate this point, the escape rates for H and D are plotted in Fig. 6 over a range of exobase temperatures. A general characteristic of these curves is that the escape rate increases quickly at low temperatures and then flattens out at higher values. To compare the escape fluxes with the available column of atoms, the number density (number per cubic meter) can be multiplied by the scale height (roughly 10^5^ cm). It can be seen that over this range of temperatures, the H escape rate is substantial, while the D escape rate is far too slow to diminish the column of atoms (Table 1). As one example, for the case of the two components with 90% at 280 K and 2% at 600 K, the increase in fraction of atoms above the escape speed is a factor of 2 for H and 50 for D atoms.

**Table 1.** Derived global escape fluxes for H and D.

Species	Season	Atmospheric column (atoms/cm^2^)	Temperature (Kelvin)	Thermal escape rate (atoms/cm^2^-s)	Thermal lifetime to escape (days)	“Hot” escape rate (atoms/cm^2^-s)*	“Hot” lifetime to escape (days)*
H	Aphelion	1.9 × 10^13^	200	8 × 10^6^	30	1.6 × 10^7^	15
	Perihelion	5.8 × 10^14^	280	1 × 10^9^	7	2 × 10^9^	3
D	Aphelion	3.8 × 10^10^	200	30	15,000	600	730
	Perihelion	6.3 × 10^11^	280	3.5 × 10^4^	200	7 × 10^5^	10
H_2_O	Aphelion	<1 × 10^14^					
	Perihelion	7 × 10^15^					

*“Hot” values refer to the case presented in the text and Fig. 3 with 2% superthermal atoms.

Evidence for hot H has been found in altitude profiles of the Lyman-α emission in data from MEX SPICAM (*62*) and HST (*29*, *63*), and D at the same elevated temperature will escape far faster than thermal D. It should be kept in mind that superthermal processes can also lead to much faster escape of molecular hydrogen in the forms H_2_ and HD. These processes must be evaluated to have a good understanding of the fractionation factor and how it relates to other processes. The presence of hot atoms, their number, and velocities are critical to determine the true fractionation factors at Mars in the present epoch and to understand the physical processes that control escape.

### Processes for the production of superthermal H and D atoms

In the text above, numbers are quoted for an assumed upper atmospheric temperature of 280 K near perihelion and a superthermal component at 600 K to be consistent with earlier calculations (*29*). This is a representative temperature, and superthermal atoms will have velocities that depend on the reaction and not be in thermodynamic equilibrium but have specific energies. Several processes have been proposed for the production of hot neutral atoms in the upper Martian atmosphere with energies well in excess of the thermal population (*62*–*64*, *84*–*86*). The main processes that have been considered for hydrogen are the following:

$$H^{+}+H--->H^{*}+H^{+}$$

$$O^{*}+H--->O+H^{*}$$

$${\text{HCO}}^{+}+e^{-}--->\text{CO}+H^{*}$$

$${{\text{CO}}_{2}}^{+}+H_{2}--->{\text{OCOH}}^{+}+H^{*}$$

$$O^{+}+H_{2}--->{\text{OH}}^{+}+H^{*}$$

where H^*^ represents a superthermal atom. Similar processes will apply to D atoms. The first reaction is the production of fast H atoms through charge exchange and collisions of neutrals with solar wind protons, and the others are collisional or chemical reactions occurring in the upper atmosphere. The relative importance of the different reactions is not well determined, although recent work indicates that the first process can fit data in which high altitude H emission profiles are dominated by a hot component (*63*). Further work should be able to constrain the relative contributions of the other processes (*64*). With observations of the bright H emission constraining the processes that lead to superthermal H atoms, the same processes can then be assumed to lead to fast D atoms to constrain the overall rate of escape of D into space.

### Atmospheric column densities and escape rates

Simple estimates have been made for the purpose of discussing the timescales for different processes related to atmospheric escape from Mars. The measurements reported in this paper are of H and D Lyman-α emissions from observations looking down on the sunlit Mars disk. In this geometry, the observed emissions come from a column of atmosphere down to ~80 km altitude, below which CO_2_ photoabsorption dominates, and most of the emission comes from altitudes 80 to 100 km. The column abundance of atoms above 80 km has been estimated by integrating with height assuming the number density at 80 km, which has been derived from the observations using the RT model. The atmospheric structure with altitude has been calculated for three cases with different assumptions about the density variations between 80 and 120 km altitude. The most realistic case is with eddy and molecular diffusion determining the H and D densities with altitude, and the density values have been checked against a more detailed model of (*83*). The overall seasonal trends are little affected by the choice of model, while the assumed case gives relatively higher column densities.

Escape fluxes have been derived from Eq. 3, and then the lifetime for a column of atoms to escape is simply the ratio of the column density to the escape rate. Global escape rates have been derived from local measurements using the assumed variations in temperature and density with SZA given in (*66*). Values are given in Table 1, where the columns are above 80 km and the temperatures and escape rates apply to the exobase at roughly 200 km altitude. The supply rate of H from upwelling water has been estimated from measured H_2_O densities at 80 km from (*45*), with an upper limit at aphelion, and then integrated with altitude assuming a mean atmospheric scale height of 15 km.

The values in Table 1 are from the representative curves in fig. S3. It is seen that for thermal processes, the column of H atoms escapes in a time that is short compared with a Martian year, while the lifetime of D atoms is much longer. Thermal escape of H is therefore limited by the diffusion of fresh atoms from the lower atmosphere, while for thermal D, it is not, requiring the added escape due to superthermal processes discussed in the text. Near aphelion, the D/H ratio is within a factor of 2 of the value found in Martian water, and the escape rate appears consistent with the slow upward diffusion of H_2_ and HD (*18*, *55*). Near perihelion, the added source of H and D atoms from water in the middle atmosphere is required to explain the high densities and escape rates.
