# Supplementary material for: Martian atmospheric hydrogen and deuterium: Seasonal changes and paradigm for escape to space
Source: Sci Adv. 2024 Jul 26;10(30):eadm7499. doi: 10.1126/sciadv.adm7499 (PMC11277398; doi:10.1126/sciadv.adm7499)
Supplement: Supplementary file 1 — Supplementary Text Figs. S1 to S5 [file sciadv.adm7499_sm.pdf]

Science Advances
AAAS

Supplementary Materials for

Martian atmospheric hydrogen and deuterium: Seasonal changes and paradigm for escape to space

John T. Clarke *et al.*

Corresponding author: John T. Clarke, jclarke@bu.edu

Sci. Adv. **10**, eadm7499 (2024)
DOI: 10.1126/sciadv.adm7499

This PDF file includes:

Supplementary Text
Figs. S1 to S5

Supplementary Materials

Measured Brightness Values for H and D Lyman-α Emissions:

In the observations of Mars the measured quantities are the brightnesses of H and D Lyman-α emissions. These emissions are produced by atomic resonant scattering of incident solar Lyman-α emission. The solar emission varies by roughly a factor of two over the solar cycle, and the solar flux at Mars varies in addition by the changing orbital distance of Mars from the Sun. The H emission is optically thick and has a relatively strong dependence on solar zenith angle which can be calculated by radiative transfer modeling. A model relation has been derived for nadir observations of points with varying solar zenith angle to relate measured values to the expected brightness at the sub-solar point. This correction is needed to compare values at different solar zenith angles. For the optically thin D emission the expected brightness is simply proportional to the density of the column of atoms, and to compare different observations the relation of density with solar zenith angle from Hodges and Johnson (*79*) has been applied. This is a relatively minor correction. For the MAVEN data no correction has been applied for the changing solar flux since these observations were all obtained around solar minimum when the solar flux is relatively constant.

The original measured brightness values and derived values at the subsolar point are plotted for hydrogen in **Figure S1** and for deuterium in **Figure S2**. As a test of the correction for H brightness the corrected values have been found to be fairly constant near aphelion, as expected from prior observations and from theory. The residual changes near perihelion are then interpreted to be real changes with time. For the D brightness values there is little change with the correction. The HST observations covered either one small region on the planet disc (GHRS) or an extended area across the disc (STIS). The HST data were obtained over a range of solar activity conditions and Lyman-α flux, and the HST values have been scaled for solar Lyman-α flux to the mean level during the MAVEN observations in the right-hand panels.

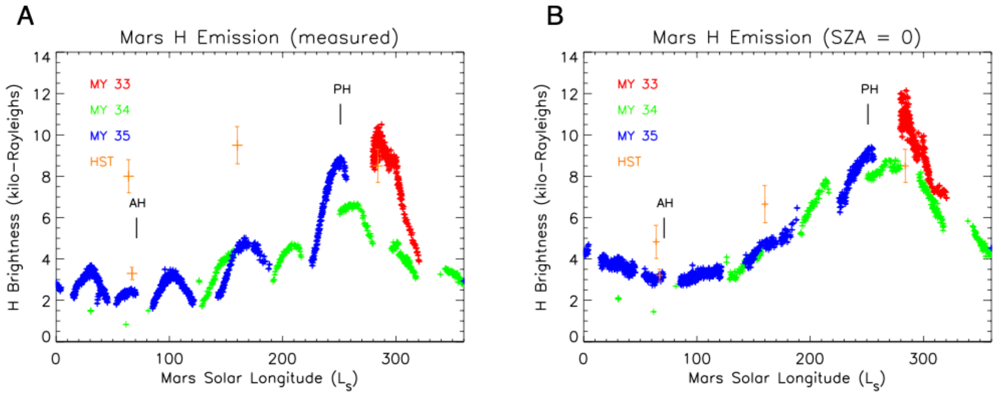

Figure S1: **Measured and corrected H brightness values**. Measured brightness values (A) and corrected to subsolar brightness (B) for H Lyman-α emission. AH and PH indicate times of aphelion and perihelion.

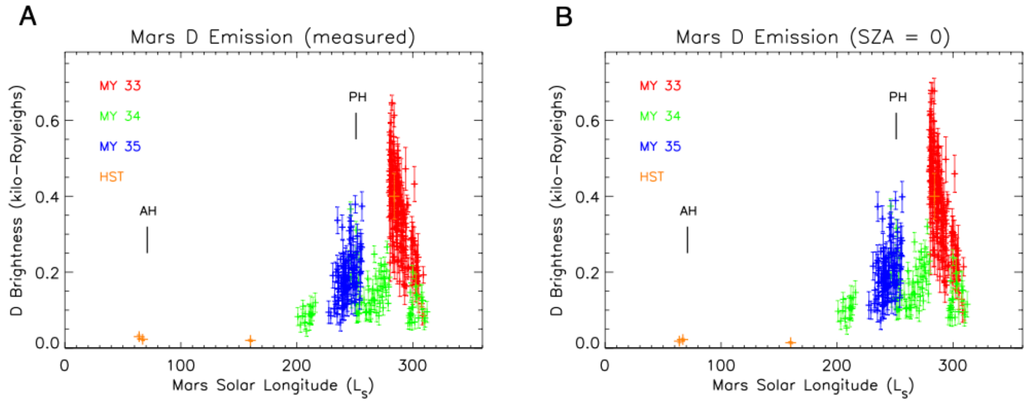

Figure S2: **Measured and corrected D brightness values.** Measured brightness values (A) and corrected to subsolar brightness (B) for D Lyman-α emission. AH and PH indicate times of aphelion and perihelion.

Fitting the Seasonal Changes in Density:

To provide functional relations for future modeling of seasonal changes, fits have been made to the density values vs. Mars solar longitude (L_S, Figure 1) employing an assumed sinusoidal function. Since there are seasonal gaps in the data and a high degree of variability near perihelion, representative curves are presented rather than detailed fitting. The seasonal trends discussed below are large compared with deviations of these representative curves from the data. The fitted values for D near aphelion have been determined by the HST measurements, since the IUVS measurements in that time range are upper limits. Since the rapid changes near perihelion do not repeat from year to year we have attempted only a time-averaged fit to the broader seasonal changes.

For the fits to the seasonal trend in the data on H and D densities with Mars solar longitude the following functional form was used consistent with prior fits in (*39*):

$$density(L_s) = 10^{A\,Sin\left(\frac{2\pi L_S}{360°}+\emptyset\right)+B} \qquad \text{(S1)}$$

where A, B, and Ø are constants, the density is expressed in units of #/cm^3 and the escape flux in units of #/cm^2-sec. The values used for the fits to the slow seasonal changes plotted in **Figure S3** are H density (A = 2.0, B = 3.95, Ø = 3.1, Y-offset = 55000), H escape flux (A = 2.3, B = 6.4, Ø = 3.1, Y-offset = 1 x 10^6), D density (A = 2.0, B = 1.38, Ø = 3.1, Y-offset = 200), and D escape flux (A = 1.2, B = 2.6, Ø = 3.1, Y-offset = 50).

The purpose of these fits is to provide representative curves that modelers may use without including the large short-term changes near perihelion, since these vary considerably from year to year. The H and D curves were assumed to have peaks coincident in time on average, although further observations may show different timings for the H and D increases. The conclusions of this paper are based on the large-scale seasonal changes, and do not rest an obtaining a better fit to the data points.

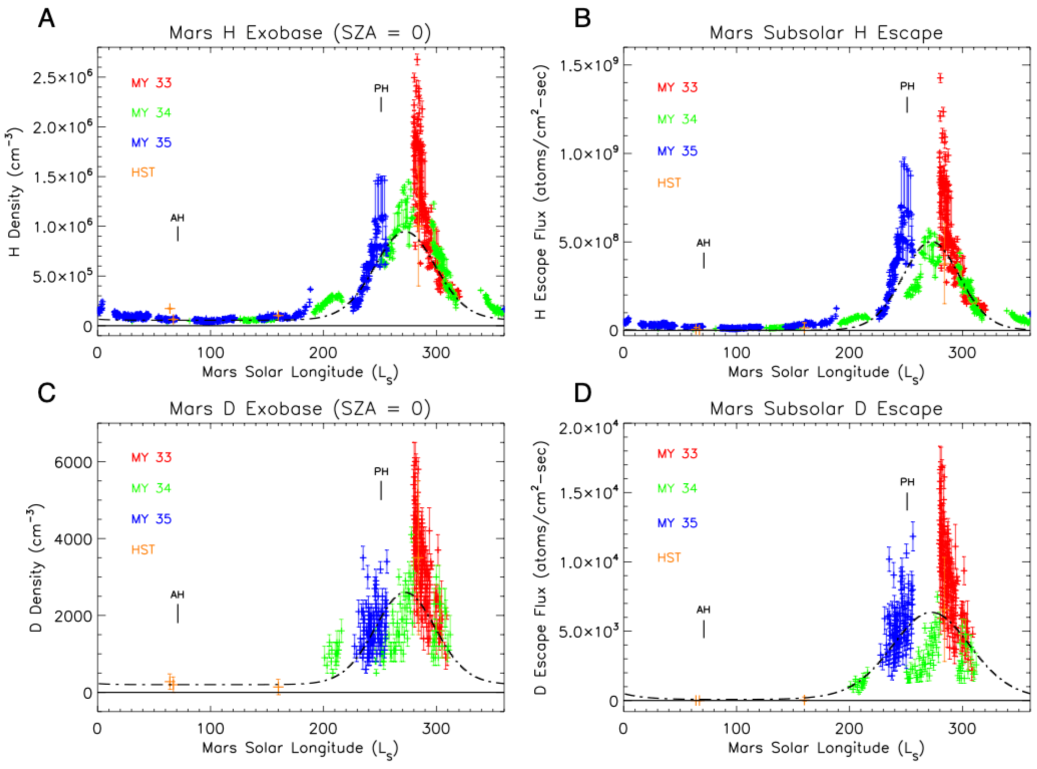

Figure S3: **Data from Figure 1 overplotted with fitted curves with the coefficients given in the text**. A) and B) are for hydrogen and C) and D) for deuterium. These are functional relations for future modeling of seasonal changes in H and D density and escape fluxes. AH indicates the time of Mars aphelion and PH indicates perihelion.

In addition, **Figure S4** gives plots of the H and D density values zoomed in on the period near perihelion to provide a better look at the time scale for changes. Finally **Figure S5** presents the values for the fractionation factor *f* with the assumption of 2 % nonthermal atoms plotted on a linear scale to show the level of variation between points. The changes in *f* are statistically significant but it is not yet known if the variation is due to location, time, etc. Further observations may better establish these relations.

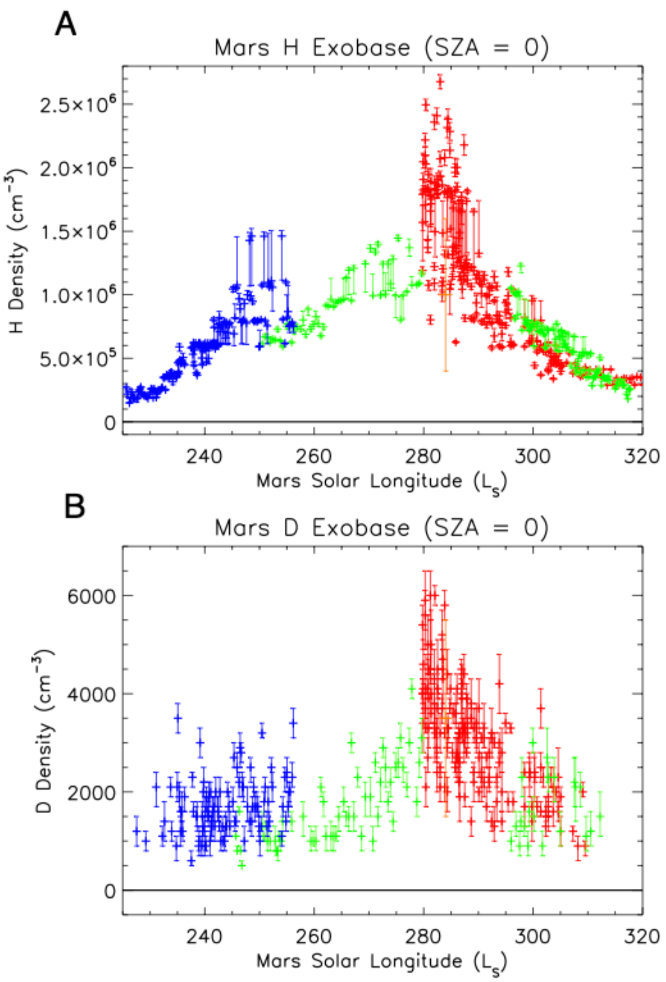

Figure S4: **Zoomed in plot of MAVEN IUVS values.** Values of H and D density of hydrogen (A) and deuterium (B) densities color coded by MY as in Figure 1 to show short-term changes near Mars perihelion (L_S 271).

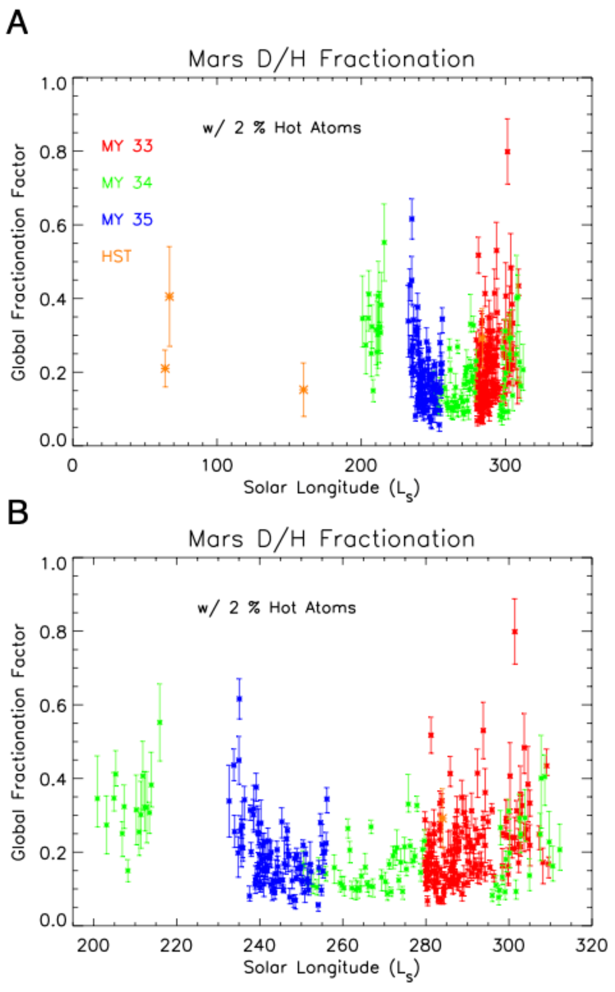

Figure S5: **The fractionation factor *f* plotted on a linear scale**. These values are calculated assuming 2 % nonthermal atoms and plotted on a linear scale to show the level of variation between points, plotted for the whole year (A) and zoomed in on perihelion / southern summer (B).
